# The role of health insurance literacy in the process and outcomes of choosing a health insurance policy in the Netherlands

**DOI:** 10.1186/s12913-023-09960-0

**Published:** 2023-09-18

**Authors:** Laurens Holst, Adriana Elisabeth Maria Brabers, Jeannette Josepha Dingena Johanna Maria Rademakers, Judith Danielle de Jong

**Affiliations:** 1 Nivel, the Netherlands Institute for Health Services Research, PO Box 1568, Utrecht, 3500 BN the Netherlands; 2 CAPHRI, Maastricht University, PO Box 616, Maastricht, 6200 MD the Netherlands

**Keywords:** Health insurance literacy, Health insurance policy, Netherlands

## Abstract

In several countries, citizens are expected to be critical consumers when choosing a health insurance policy. However, there are indications that citizens do not always have the sufficient skills, so called health insurance literacy (HIL), to do this. We investigated whether the level of HIL among Dutch citizens is related to the way in which they experience the process of choosing a policy, and furthermore whether it is related to their health insurance choices. We obtained information by sending questionnaires to members of the Nivel Dutch Health Care Consumer Panel in 2020. Of the 1,500 approached, 806 panel members participated (response rate 54%). Our results indicate that, compared to those with a high HIL, respondents with a low HIL more often find choosing a health insurance policy difficult, not interesting, and boring, and less often consider it important and worthwhile. Furthermore, they make less use of the opportunity to switch from one health insurer to another. However, they do still opt for a supplementary insurance policy and a voluntary deductible to the same extent as citizens with a high HIL. We conclude that the HIL level among Dutch citizens is related to the way in which they experience the process of choosing a health insurance policy and to the extent to which they switch from one insurer to another. But it is not related to their health insurance choices. Follow-up research should focus on how citizens with a low HIL can be better supported when choosing a health insurance policy.

**Supplementary Information:**

The online version contains supplementary material available at 10.1186/s12913-023-09960-0.

## Introduction

Several Western countries, such as the United States, Switzerland, Israel, Germany, and the Netherlands, have health insurance systems in which citizens are expected to be critical consumers when choosing a health insurance policy [1–5]. Citizens from these countries have, within a certain period or after a certain period of time, the opportunity of choosing with which insurer they want to be insured and how they want to be insured for the upcoming period. This possibility for citizens to switch from one health insurer to another should stimulate insurers to continue providing good care and service, and to offer good policies at a competitive premium [6, 7]. If citizens do not act as critical consumers, and do not make well-informed decisions with regard to their health insurance policy, there is a chance that they will be sub-optimally insured [8–10]. This may have financial consequences [11]. On the one hand, these citizens may lack sufficient insurance cover and might have to deal with unexpected costs. On the other hand, they may take out more insurance than is necessary, resulting in them paying a higher premium than they need to, or are insured for costs they are not likely to make. In addition to these financial consequences for the individual citizens, a lack of a critical attitude toward health insurance policies can also have negative consequences for the functioning of the system as a whole. If too few citizens make use of their opportunity to switch, insurers may not be sufficiently stimulated to continue to offer good health policies at a competitive premium [8, 12].

There are many indications that not all citizens are able, or willing, to act as critical consumers and make informed decisions when choosing a health insurance policy [11, 13]. One reason for this is the complexity of the health insurance system with a large number of policies to choose from [12, 14, 15]. Citizens may be overwhelmed when trying to find a suitable policy. This idea is supported by the theory of ‘Decision Avoidance’ described by Anderson as: ‘a tendency to avoid making a choice by postponing it or by seeking an easy way out that involves no action or no change’ [16] p. 139). Furthermore, there is evidence that many citizens do not find choosing a health insurance policy interesting enough, making them less inclined to delve into the possible options regarding the choices for a policy [17–19]. As a consequence, they do not take the opportunity to switch health insurers. In addition to the fact that citizens may be overwhelmed or uninterested, there is also evidence that citizens do not always have sufficient skills to choose a suitable health insurance policy [20, 21]. These skills are called Health Insurance Literacy (HIL), and can be described as ‘*the extent to which consumers can make informed purchase and use decisions regarding health insurances’* [22]. HIL is related to the concept of health literacy which refers to people’s skills to access, understand, assess, and act upon health-related information [23], in this case about health insurance policies.

Studies focusing on HIL mostly relate to the North American health insurance market [24]. Several studies have shown that US citizens with a limited HIL have more difficulty choosing and taking out a policy [11, 25–27]. It seems that citizens with a limited HIL are less able to find their way to a suitable health insurer. This emphasizes that it is also valuable to investigate the concept of HIL in other countries in which citizens are expected to fulfil an active role in choosing a health insurance policy. In this study, we focus on the Dutch health insurance system. Here, citizens are obliged to have a health insurance policy, and they have the possibility of choosing a different basic health insurance policy with a different insurer every year. In 2020, for example, citizens could choose from 55 basic policies with 21 private health insurers [28]. In addition, they can also opt for a voluntary deductible each year (an optional increase of the amount that citizens must pay out of pocket before an insurer reimburses the costs, thereby reducing the premium) and may consider too taking out a supplementary insurance policy (policies that provide coverage for additional healthcare services such as dental care and physiotherapy). To date, little is known about the HIL of citizens in the Netherlands. A recent study showed that Dutch citizens who had less education or earned a lower income have relatively more difficulty in choosing and using a health insurance policy [29]. The findings of this study align with what it is known about health literacy, namely that people who are less advantaged due to their socio-economic position have more difficulty accessing, understanding, appraising, and using health-related information than people who are more advantaged [30, 31].

It is known that the level of education and income is related to the HIL of citizens in the Netherlands. However, there is still too little knowledge of the extent to which HIL has an impact upon the process of selecting a health insurance policy. It may be possible that citizens with a lower HIL, compared to those with a higher HIL, are less engaged in choosing a policy because the health insurance system and the information about insurances is too complex for them. Consequently, they might switch less from one health insurance to another. In addition, it could also be that citizens with a lower HIL are insured in a different way, for example with less comprehensive cover, than those with a higher HIL. In order to gain more insight into these questions, we focused, in this study, on the role of HIL in the process and outcomes of choosing a health insurance policy in the Netherlands. We investigated whether the HIL level of citizens is related to the way in which they make decisions regarding their health insurance policy, and consequently to the way in which they are insured. We answered the following research questions:

- Does the HIL level affect the experience of choosing a health insurance policy?
- How does the HIL level relate to the behaviour of citizens in switching their health insurance policy?
- How does the HIL level affect the choice of health insurance?

In this way, more insight is gained into the extent to which citizens can fulfill their role as critical consumers in choosing a health insurance policy, as is expected of them in the Dutch health insurance system.

## Materials and methods

In February 2020 Nivel (the Netherlands Institute for Health Services Research), an independent foundation which contributes to the quality and effectiveness of the Dutch healthcare system, sent its annual monitor investigating ‘switching health insurer’ to 1,500 members of the Dutch Health Care Consumer Panel (DHCCP).

### Study population

The DHCCP is an access panel [32], which consists of a large number of individuals who have agreed to answer questions related to healthcare on a regular basis. At the time of this study, the panel consisted of approximately 12,000 members of whom various demographic characteristics were known, such as gender, age, and level of education. The panel is regularly renewed to ensure that representative samples of the Dutch population can continue to be drawn. Nivel recruits possible new members by buying address files from address suppliers. As a result, possible new members for the panel are sampled at random from the general population in the Netherlands. If they want to become a member, they can fill in an introduction questionnaire with questions about their characteristics. By returning thisquestionnaire respondents are considered to have given consent to participate in studies within the panel. Furthermore, they have been made aware that their answers could be used for multiple research purposes. There is no possibility of individuals signing up for the panel on their own initiative.

As previously described in the study by Holst et al. [29], which also made use of the DHCCP and of the dataset concerning the annual monitor of ‘switching health insurer’, all data were collected and processed in accordance with the privacy policy of the DHCCP. The panel complies with the General Data Protection Regulation (GDPR). According to Dutch legislationapproval by a medical ethics committee is not obligatory for conducting research through this panel. Participation was voluntary. Panel member were free to answer the questions or not. All methods in this study were carried out in accordance with relevant guidelines and regulations.

### The monitoring of ‘switching health insurer’

With the annual monitor of ‘switching health insurer’ Nivel investigates each year, among other things, what kind of health insurance policy citizens have chosen for the upcoming year, whether they have switched between health insurers, and if so, what are their reasons for switching. A multidisciplinary research team including experts in the Dutch healthcare system - among whom were two co-authors of this study, JdJ and AB - developed the questions for the monitor. Different representatives from the healthcare sector who sit on the programme committee of the DHCCP commented upon the draft version of the monitor. These included the Dutch Ministry of Health, Welfare and Sport, the Dutch Consumers Association, the Netherlands Patients Federation, and the umbrella organisation of health insurers in The Netherlands, Zorgverzekeraars Nederland.

The monitor was sent out using a mixed-mode method that is either by post or through the internet depending on the panel member’s preference. The 1,500 panel members who were approached were representative of the adult population in the Netherlands (18+) regarding gender and age (stratified sampling). In order to generate the largest possible response, a number of reminders were sent to the respondents who had not yet completed the questionnaire. Table A1 in the appendix provides an overview of the questions extracted from the monitor in order to answer the research questions of this study.

### Health insurance literacy measure in the Netherlands (HILM-NL)

The HILM-NL questionnaire was used to measure the HIL among citizens in the Netherlands. The HILM-NL is a self-assessment measure of the ability of Dutch citizens to choose a health insurance policy, and to use it once enrolled [33]. The HILM-NL questionnaire, the Dutch version of the original US Health Insurance Literacy Measure [34], which has been translated and validated in Dutch according to the WHO guidelines for translation and adaption of instruments [33], was also part of Nivel’s ‘switching health insurer’ monitor in February 2020. The HILM-NL questionnaire has proven to be a valid and reliable instrument [35]. The measure is publicly available on the Nivel website: https://www.nivel.nl/nl/publicatie/health-insurance-literacy-measurement-nederlands-hilm-nl.

As previously described in the study by Holst et al. [29], which also made use of the HILM-NL questionnaire, it consists of twenty-one questions that are categorised into four subscales. These are: (1) confidence in choosing a health insurance policy (six questions); (2) behaviour in choosing a health insurance policy (seven questions); (3) confidence in using a health insurance policy (four questions), and; (4) behaviour in using a health insurance policy (four questions). The four subscales can be grouped into two domains, “confidence” (subscale 1 and 3), and “behaviour” (subscale 2 and 4). Answers to the questions of the domain “confidence” are scored on four-point ordinal scales. These are: not at all confident (1); slightly confident (2); moderately confident (3), and; very confident (4). Answers to the questions of the domain “behaviour” are also scored on four-point ordinal scales. These are: not at all likely (1); somewhat likely (2); moderately likely (3), and; very likely (4). As with the HILM developed in the US, respondents are excluded if they answer fewer than three questions on subscale 1 and 2, or fewer than two on subscale 3 and 4.

The total HILM-NL score, as well as the score per subscale or domain, can then be calculated on the basis of the categories. Since the questionnaire contains a total of twenty-one questions, the total HILM-NL score can range from 21 (marked one on all questions) to 84 (marked four on all questions). Based on the distribution of the same data analysed in a previous study [29], we consider citizens with a HILM-NL score below 50 as having low health insurance literacy, citizens with a HILM-NL score between 50 and 60 as having intermediate literacy, and those with a HILM-NL score higher than 60 as having high literacy. Higher scores imply a higher self-assessed ability in selecting and using health insurance.

### Demographic characteristics

The following demographic characteristics were included: gender, age, level of education, household net income per month, self-reported health, and self-reported amount of care used. There is no multicollinearity between these characteristics. Gender was divided in two categories (1 = man, 2 = woman). Age was divided in three categories (1 = 18–39 years, 2 = 40–64 years, 3 = 65 years and older). The level of education was classified as: low (none, primary school or pre-vocational education) (1); intermediate (secondary or vocational education) (2), or; high (professional higher education or university) (3). The household net income per month was classified as: low (less than 1,750 euro) (1); intermediate (between 1,750 and 2,700 euro) (2), or; high (more than 2,700 euro) (3). For the variable ‘self-reported health’, the panel members participating were asked how they rank their general health using the SF-36, a frequently used valid and reliable instrument for measuring self-perceived health [36]. They could choose between five options: bad, fair, good, very good, or excellent. We classified the self-reported health as: bad (bad or fair) (1), good (good) (2), or very good (very good or excellent) (3). For the variable ‘self-reported amount of care used’, the panel members participating were asked how much they think they make use of healthcare. They could choose between five options: none, very little, little, much, or very much. We classified the self-reported amount of care used as: none (none) (1), little (very little or little) (2), or much (much or very much) (3).

### Statistics

Each figure or table shows the number of respondents (n) who answered the question. The numbers differ per question because the respondents did not always answer all the questions. For each question of table A1 in the appendix, a chi-square test was used to assess whether the HIL level was associated with the way the question was answered. Assumptions (the data was categorical and consisted of two or more independent groups) for performing chi-square tests were met. To gain a better understanding of the association of HIL with the process and outcomes regarding the choice of a health insurance policy, logistic regression analyses were performed. In these analyses the demographic characteristics are included as covariates to correct for nonresponse bias. Based on the assumptions for performing logistic regression analysis, a dichotomous - (Q5-Q8 in table A1), an ordinal - (Q1,Q3,Q4 in table A1), or a multinomial logistic regression analysis (Q2 in table A1) was used. Only significant results found in relation to the HIL score, are reported in the results section. A significance level of 5% (p ≤ 0.05) was used for the analyses in this study. All analyses were performed with STATA version 16.1.

## Results

A total of 806 of the 1,500 panel members approached completed the questionnaire, a response rate of 54%. Twenty-five respondents were excluded because they answered too few questions on the HILM-NL. The results in this study are, therefore, based on 781 respondents.

### Health insurance literacy measure in the Netherlands (HILM-NL)

As described before, the total group was divided into three. Thirty-two per cent of the respondents who had a HILM-NL score below 50 and were considered as having low health insurance literacy, 33% with a HILM-NL score between 50 and 60 were considered having intermediate health insurance literacy, and 35% who had a HILM-NL score higher than 60 were considered to have high health insurance literacy.

### The demographics of the respondents

Table 1 shows that the gender of the respondents was nearly evenly distributed (49%/51%) and 16% were between the ages of 18 and 39. Slightly more than one in ten respondents (11%) reported a low level of education and slightly less than a quarter (23%) reported a net monthly household income lower than 1,750 euro. Almost one in five respondents (19%) indicated their health was bad or fair and 7% indicated that they consumed no care.

**Table 1:** The demographics of the respondents

		n	%
Total		781	
Gender	Male	386	49
	Female	395	51
Age	18–39	123	16
	40–64	424	54
	65 and older	234	30
Highest completed education level*	Low	85	11
	Intermediate	352	45
	High	333	43
	Unknown	11	1
Household net income per month in euros	< 1.750	176	23
	1.750–2.700	232	30
	> 2.700	343	44
	Unknown	30	4
Self-reported health	Bad / fair	147	19
	Good	392	50
	Very good / excellent	216	28
	Unknown	26	3
Self-reported amount of care used	None	58	7
	Very little / little	516	66
	Much / very much	184	24
	Unknown	23	3

* Low = none, primary school or pre-vocational education. Intermediate = secondary or vocational education. High = professional higher or university

### Does the HIL level affect the experience of choosing a health insurance policy?

**Fig. 1:**
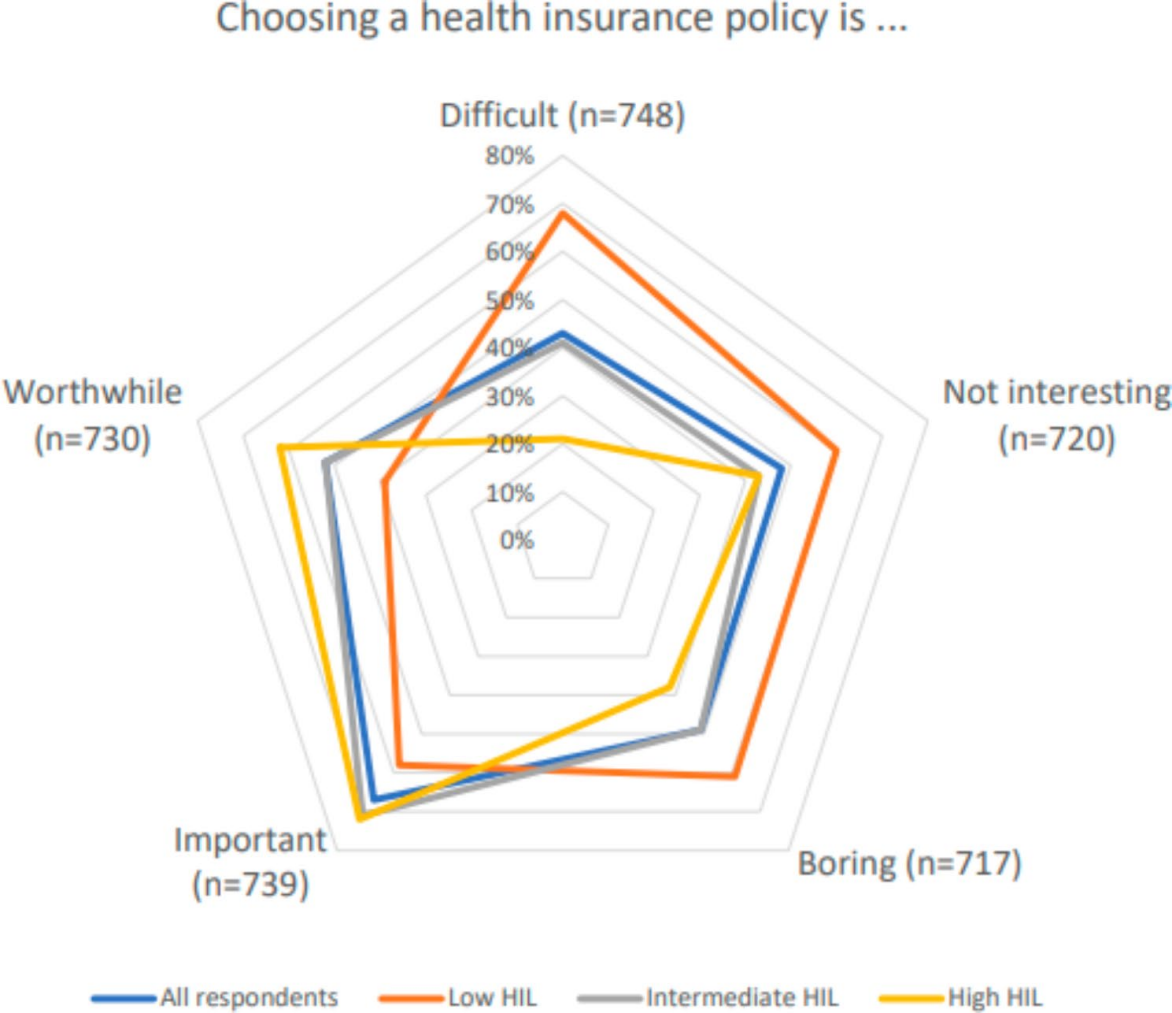
What do you think about choosing a health insurance policy? (n = 717–748). * Figure 1 shows the percentages of respondents that choose score 1 or 2 out of a ordinal scales with a range from 1 to 5

Figure 1 shows, among other things, how respondents with low, intermediate, and high HIL experience choosing a health insurance policy. Among respondents with a low HIL, 68% indicate that it is difficult, compared to 21% among those with a high HIL (*X*^*2*^, *p < 0.01*). In addition, Fig. 1 shows that 60% of the respondents with a low HIL did not find choosing health insurance interesting, 61% thought it was boring, 58% thought it was important, and 39% thought it worthwhile. Among respondents with a high HIL, these percentages are respectively 43%, 38%, 72%, and 62% (*X*^*2*^, *p < 0.01 for all*). Ordinal logistic regression analyses (see table A2 in the appendix), were performed to correct for the demographic characteristics we included. These confirmed that there is an association between the level of HIL, be it high or low, and the extent to which respondents indicate that they find such a choice difficult, not interesting, boring, important, and worthwhile *(p < 0.01 for all)*.

**Fig. 2:**
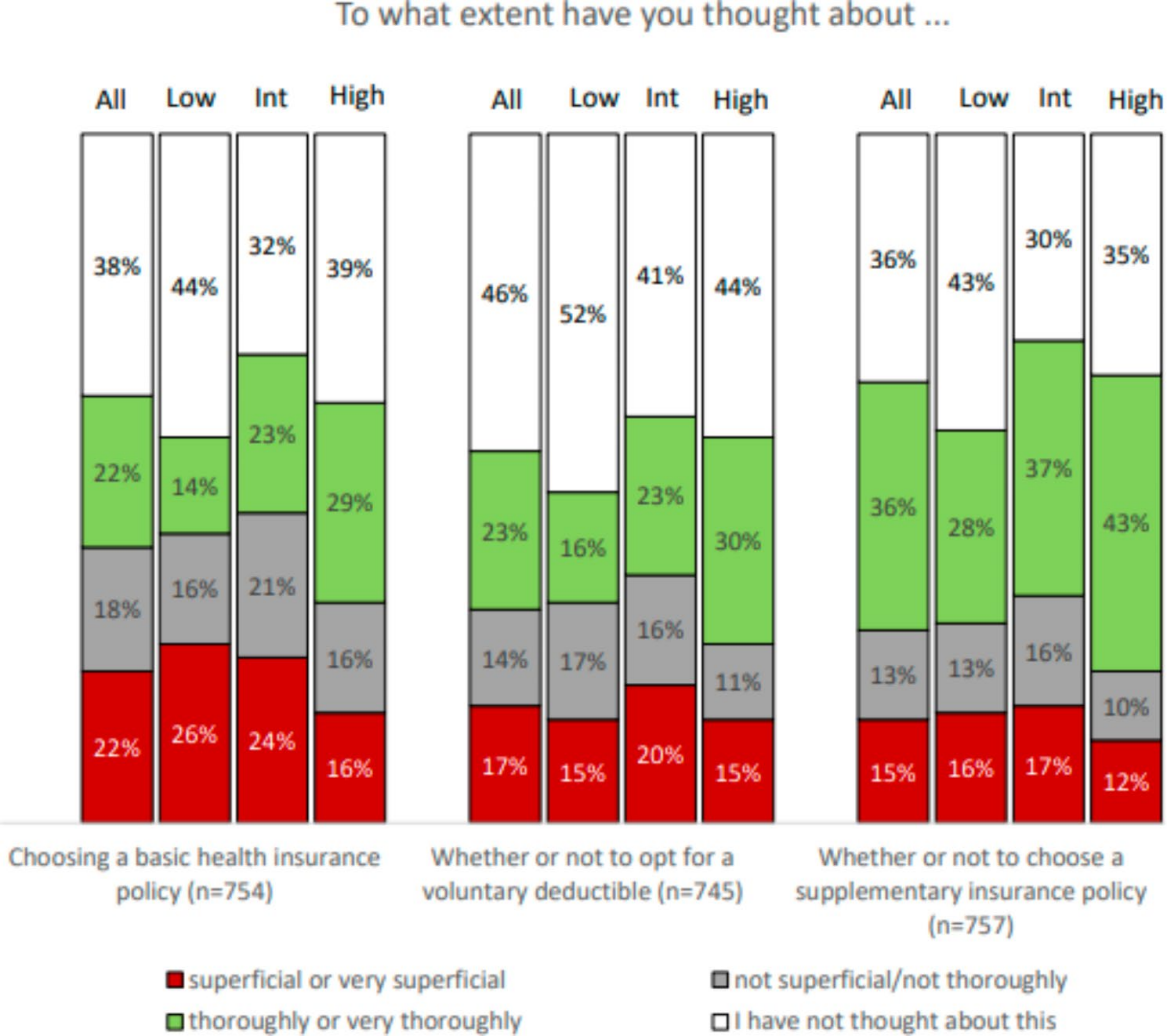
Thinking about health insurance options (n=745-757) * All = all respondents, Low = respondents with a low HIL, Int = respondents with an intermediate HIL, High = respondents with a high HIL

Figure 2 shows, among other things, the extent to which respondents with low, intermediate, and high HIL have thought about their health insurance options. Fewer respondents with a low HIL indicated that they thought thoroughly, or very thoroughly, about choosing a basic health insurance policy (14%), whether or not to opt for a voluntary deductible (15%), and whether or not to choose a supplementary insurance policy (28%). This compares to respondents with a high HIL whose percentages were respectively 29%, 30%, and 43%) (*X*^*2*^, *p < 0.01 for all aspects*). Multinomial logistic regression analyses (see table A3.1-A3.3 in the appendix) confirm that there is an association between high - versus low HIL and the extent to which respondents indicate that they have thought about these aspects *(p < 0.05 for all)*.

Furthermore, we presented the following statements to the respondents: 1) I spend a lot of time looking for the right information about health insurance policies, and; 2 ) I would like more help with how to find the right information about health insurance policies. Compared to those with a high HIL, fewer respondents with a low HIL agree, or totally agree, with the first statement (19% vs. 34%). In addition, more respondents with a low HIL agree, or totally agree with the second statement (36% vs. 13%) (*X*^*2*^, *p < 0.01 for both statements*). Ordinal logistic regression analyses (see table A4 in the appendix) confirm that there is an association between high - versus low HIL and the extent to which respondents agree with the statements *(p < 0.01 for both statements)*.

**Fig. 3:**
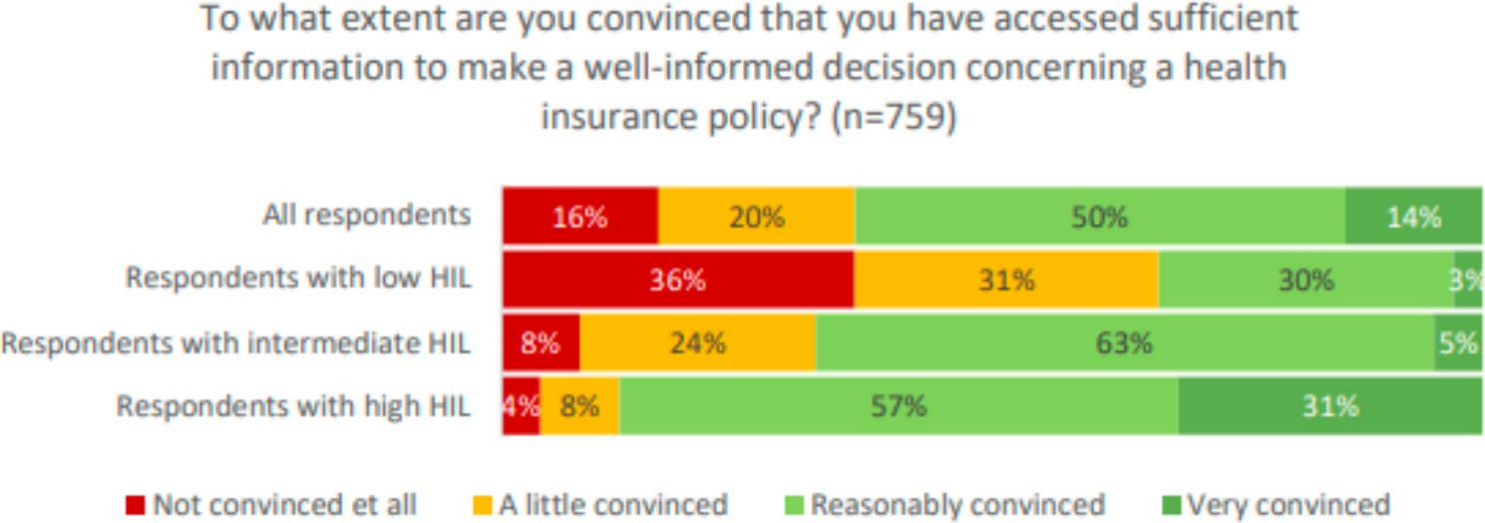
Confidence in the amount of information accessed concerning health insurance policies

Figure 3 shows that fewer respondents with a low HIL (33%) indicated that they are reasonably, or very, convinced that they have accessed sufficient information to make a well-informed decision concerning a health insurance policy, compared to respondents with a high HIL (88%) (*X*^*2*^, *p < 0.01*). Ordinal logistic regression analyses (see table A5 in the appendix) confirmed that there is an association between high - versus low HIL and the extent to which respondents indicated that they are convinced they have enough information *(p < 0.01)*.

Our results show that, compared to respondents with a higher HIL, those with a lower HIL indicate more often that choosing a health insurance policy is difficult, not interesting, and boring. They indicate less often that it is important and worthwhile. Furthermore, they indicate more often that they did not think thoroughly about choosing a health insurance policy, whether or not to opt for a voluntary deductible, or whether or not to choose a supplementary insurance policy. Also, they indicate less often that they spend a lot of time looking for the right information about health insurance policies, and more often that they would like additional help with how to find the right information about such policies. Finally, they indicate less often that they are convinced that they have accessed sufficient information to make a well-informed decision concerning a health insurance policy.

### How does the HIL level relate to the behaviour of citizens in switching their health insurance policy?

6% of the respondents indicated that they had switched for the upcoming year (2020), and 75% of the respondents indicated that they had not switched health insurers in the past five years. When we compare groups it stands out that among those with a low HIL, 4% indicated that they have switched in 2020, and 80% indicated that they had not switched in the past five years. Among those with a high HIL, 10% indicated that they have switched, and 70% indicated that they had not switched in the past five years (*X*^*2*^, *p < 0.05 for both questions*). Multivariate logistic regression analyses (see table A6 in the appendix), performed to correct for demographic characteristics, confirm that there is an association between high - versus low HIL and the extent to which respondents indicate that they have switched *(p < 0.05 for both questions)*.

Our results show that, compared to respondents with a higher HIL, respondents with a lower HIL switch less often from one health insurer to another.

### How does the HIL level affect the choice of health insurance?

Chi square tests and multivariate logistic regression analyses (see table A7 in the appendix) showed no association between the level of HIL and the extent to which respondents had a voluntary deductible or a supplemental insurance policy (*p > 0.05 for both questions*). Among the respondents, 88% of those with a low HIL indicate that they have opted for a supplemental insurance policy compared to 84% of respondents with a high HIL. In addition, 15% of respondents with a low HIL indicate that they have opted for a voluntary deductible compared to 16% of respondents with a high HIL.

Our results therefore show that respondents with a low HIL opt for a supplementary insurance policy and a voluntary deductible to the same extent as respondents with a high HIL.

## Discussion

The aim of this study was to investigate whether the level of HIL among citizens in the Netherlands is related to the way in which they make decisions regarding their health insurance policy, and consequently to their choice of insurance. Our results indicate that respondents with a low HIL experience the process of choosing a health insurance policy differently than citizens with a high HIL. They find choosing a health insurance policy more often difficult, not interesting, and boring, and less often consider it important and worthwhile. Also, they think less thoroughly about their health insurance options. Furthermore, our results show that respondents with a low HIL make less use of the opportunity to switch from one health insurer to another. However, they do opt for a supplementary insurance policy and a voluntary deductible to the same extent as citizens with a high HIL.

Our results are in line with previous findings regarding health literacy and related concepts, such as ‘patient activation’, which can be defined as having the knowledge, skills, confidence, and behaviours needed for managing one’s own health and healthcare [37]. From studies focusing on health literacy, it is known that individuals with a low health literacy have more difficulty evaluating whether health-related information is useful [38, 39]. Our study shows that citizens with a low HIL less often indicate that they are convinced that they have accessed sufficient information to make a well-informed decision concerning a health insurance policy. In addition, studies focusing on health literacy have shown that the extent to which someone is health literate depends not only on an individual’s cognitive skills, but also on their degree of motivation [23]. Citizens with a lower health literacy are less motivated to acquire new knowledge [40]. In a Dutch study using the patient activation measure, it further became clear that less activated healthcare consumers are less likely to seek and use health information [41]. Our results show that citizens with a low HIL less often indicate that they spent a lot of time looking for the right information about health insurance policies compared to those with a high HIL.

An important question based on our results is to what extent it is problematic that citizens with a low HIL are less inclined to delve into the selection process of health insurance and, indeed, are less inclined to switch their policy compared with citizens with a high HIL. After all, respondents with a low HIL opt for a supplementary insurance policy and a voluntary deductible to the same extent as respondents with a high HIL. However, the main point is not the actual outcome, but how the outcome came about. Our results suggest that citizens with a low HIL find the selection process difficult, and, as a result, may not be able to make informed decisions. This can lead to an outcome which is not in line with their actual needs and preferences, and may ultimately have financial consequences. Our results show that two thirds of the respondents with a low HIL indicate that choosing a health insurance policy is difficult, compared to one fifth of the respondents with a high HIL. Furthermore, one third of the respondents with a low HIL want to have additional help on how to find the right information about health insurance policies, compared to one eighth of the respondents with a high HIL. This is consistent with the results of a previous US study that showed that citizens with a low HIL had a greater desire to receive help in choosing a health insurance policy than those with a high HIL [27]. It seems that the provision of information about health insurance policies does not always match the skills of citizens with a low HIL.

In this study, we focus on citizens’ skills in choosing a health insurance policy. However, there are also other reasons why citizens may not be inclined to delve into the possible options regarding their choices for a policy, and consequently to make use of the opportunity to switch health insurers. Our results show that there is also a group of citizens who are simply not interested in choosing an insurance policy. It is important to take this perspective into account when assessing the results of the current study, and to interpret it in our suggestions for further research. Some citizens, regardless of whether they have a high, or low, HIL, will not have a need for support when choosing health insurance.

### Further research

Further research should focus on how citizens with a low HIL, who would like to receive support, can be guided better when choosing a health insurance policy. A better understanding of the barriers they experience during this process, and of their needs and preferences, is required. It is, for example, relevant to investigate how they could best be helped in choosing a health insurance policy, how they want to receive or look up information and who should provide it. More tailored and easily understandable information should be available to all citizens but would be especially helpful for those with a low HIL. In addition, we recommend investigating whether citizens with a low HIL make poor choices more often with regard to their health insurance policy, compared to those with a high HIL. It may be useful to evaluate whether the choice of a policy has, ultimately, been suitable or not. In addition, it is important to investigate further whether having such an unsuitable insurance policy has financial and healthcare consequences. With greater clarity it would be possible to emphasize more soundly the importance of making well informed decisions regarding health insurance policies.

### Strengths and limitations

The data from the annual monitor ‘switching health insurer’ was collected using a mixed- mode method. The respondents could complete the monitor on paper or online, based on their own preference. As a result, we were able to also collect data from respondents who are less digitally skilled. We consider the response to be reasonable (n = 806, response rate 54%). The topics within the panel invariably focus on healthcare, so it can be assumed that the panel members we approached, and those who participated, have an above-average interest, and most likely, knowledge about topics relating to health. It could, therefore, be possible that, despite approximately half of all respondents in the current study already indicating that choosing an insurance policy is not interesting and is difficult, these percentages may, in reality, be higher. This could be explored further. Finally, because research on HIL is still in its early stages in the Netherlands, it is not yet clear what level of HIL Dutch citizens should have in order to choose and use their health insurance policy properly. We are, therefore, unable to determine whether citizens are insufficiently or sufficiently skilled in making informed decisions with regard to their health insurance policy. We can only speak of low, intermediate, and high HIL.

## Conclusion

The level of HIL among Dutch citizens is related to the way they experience the process of choosing a health insurance policy, and to the extent to which they switch from one health insurer to another, but not to their health insurance choices.

### Electronic supplementary material

Below is the link to the electronic supplementary material.

Supplementary Material 1

## Data Availability

The minimal anonymized data set is available upon request from prof. Judith D. de Jong (j.dejong@nivel.nl), project leader of the Dutch Health Care Consumer Panel, or the secretary if this panel (conusmentenpanel@nivel.nl). The Dutch Health Care Panel had a program committee, which supervises processing the data of the Dutch Health Care Consumer Panel and decides about the use of the data. This program committee consists of representatives of the Dutch Ministry of Health, Welfare and Sport, the Health Care Inspectorate, Zorgverzekeraars Nederland (Association of Health Care Insurers in the Netherlands), the National Health Care Institute, the Federation of Patients and Consumer Organisations in the Netherlands, the Dutch Healthcare Authority and the Dutch Consumers Association. All research conducted within the Consumer Panel has to be approved by this program committee. The committee assesses whether a specific research fits within the aim of the Consumer Panel, which is to strengthen the position of the health care user.

## References

[1] Blümel M (2020). Germany: Health System Review. Health Syst Transit.

[2] De Pietro C et al. Switzerland: Health System Review. Health Syst Transit. 2015;17(4):1–288,xix. 26766626

[3] Kroneman M (2016). Netherlands: Health System Review. Health Syst Transit.

[4] Rice T (2020). United States: Health System Review. Health Syst Transit.

[5] Rosen B, Waitzberg R, Merkur S (2015). Israel: Health System Review Health Syst Transit.

[6] Duijmelinck DMID, Mosca I, van de Ven WPMM (2015). Switching benefits and costs in competitive health insurance markets: a conceptual framework and empirical evidence from the Netherlands. Health Policy.

[7] Thomson S (2013). Statutory health insurance competition in Europe: a four-country comparison. Health Policy.

[8] ACM Beter kiezen op de polismarkt (Make better choices in the health insurance market) Retrieved (August 2022) from: https://www.acm.nl/sites/default/files/documents/2018-07/acm-nza-rapport-beter-kiezen-op-de-polismarkt.pdf.2018

[9] van der Hulst FJP (2022). To what degree are health insurance enrollees in the Netherlands aware of the restrictive conditions attached to their policies?. Health Policy.

[10] van Winssen KP, van Kleef RC, van de Ven WP (2016). The demand for health insurance and behavioural economics. Eur J Health Econ.

[11] Bhargava S, Loewenstein G (2015). Choosing a Health Insurance Plan: complexity and consequences. JAMA.

[12] de Jong J et al. Het functioneren van de zorgverzekeringsmarkt: een kennissynthese (the functioning of the health insurance market: a knowledge synthesis). Retrieved (August 2022) from: https://www.nivel.nl/nl/publicatie/het-functioneren-van-de-zorgverzekeringsmarkt-een-kennissynthese.2015

[13] Bartholomae S (2016). Building Health insurance literacy: evidence from the Smart Choice Health Insurance™ Program. J Fam Econ Issues.

[14] McWilliams JM (2011). Complex Medicare advantage choices may overwhelm seniors—especially those with impaired decision making. Health Aff.

[15] Parragh ZA, Okrent D. Health Literacy and Health Insurance Literacy: Do Consumers Know What They Are Buying? 2015.

[16] Anderson CJ. The psychology of doing nothing: forms of decision avoidance result from reason and emotion. Psychol Bull. 2003;129:139–67. 10.1037/0033-2909.129.1.139 12555797

[17] NZa. Ruimte voor onderscheid tussen zorgverzekeraars (Room for distinction between health insurers). Retrieved (August 2022) from: https://www.acm.nl/sites/default/files/old_publication/publicaties/17402_rapport-acm-nza-ruimte-voor-onderscheid-tussen-zorgverzekeraars-03072017.pdf 2017.

[18] Looijenga M. Customer Centricity and Transparancy in the Healthcare Insurance Sector. Retrieved (August 2022) from: https://edepot.wur.nl/395221. 2016

[19] RVZ, De stem van verzekerden (the voice of the insured) Retrieved (August. 2022) from: file:///H:/Downloads/De_stem_van_verzekerden.pdf. 2014.

[20] Barnes AJ, Hanoch Y (2017). Knowledge and understanding of health insurance: challenges and remedies. Isr J Health Policy Res.

[21] Loewenstein G (2013). Consumers’ misunderstanding of health insurance. J Health Econ.

[22] Kim J, Braun B, Williams AD (2013). Understanding Health insurance literacy: a Literature Review. Family and Consumer Sciences Research Journal.

[23] Sørensen K (2012). Health literacy and public health: a systematic review and integration of definitions and models. BMC Public Health.

[24] Quiroga Gutiérrez AC. Health insurance literacy assessment tools: a systematic literature review. J Public Health (Berl.). 2023;31:1137–50.

[25] Barnes AJ, Hanoch Y, Rice T (2015). Determinants of coverage decisions in health insurance marketplaces: consumers’ decision-making abilities and the amount of information in their choice environment. Health Serv Res.

[26] Greene J, Peters E (2009). Medicaid consumers and informed decisionmaking. Health Care Financ Rev.

[27] Hero JO (2019). Decision-making experiences of consumers choosing individual-market Health Insurance Plans. Health Aff (Millwood).

[28] Vektis. Zorgthermometer: Verzekerden in Beeld 2020 (Healthcare thermometer: Insured in 2020). Retrieved (November 2022) from: https://www.vektis.nl/uploads/Publicaties/Zorgthermometer/Zorgthermometer%20Verzekerden%20in%20Beeld%202020.pdf

[29] Holst L et al. Measuring health insurance literacy in the Netherlands – First results of the HILM-NL questionnaire. Health Policy. 2022;126(11):1157-62. 10.1016/j.healthpol.2022.09.001 36180280

[30] Sørensen K (2015). Health literacy in Europe: comparative results of the european health literacy survey (HLS-EU). Eur J Public Health.

[31] van der Heide I (2013). Health literacy of dutch adults: a cross sectional survey. BMC Public Health.

[32] Brabers A, de Jong J. Nivel Consumentenpanel Gezondheidszorg: basisrapport met informatie over het panel 2022 (the Nivel Dutch Health Care Consumer Panel : basic report with information about the panel 2022). Retrieved (August 2022) from: https://www.nivel.nl/nl/publicatie/nivel-consumentenpanel-gezondheidszorg-basisrapport-met-informatie-over-het-panel-2022. 2022.

[33] Holst L et al. Health Insurance Literacy Measurement: vertalen en cultureel valideren van een meetinstrument voor de ‘zorgverzekeringsvaardigheden’ van verzekerden in Nederland (translating and cultural validation of a measuring instrument for the ‘health insurance literacy’ of insured in the Netherlands) Utrecht, Nivel, 2019.

[34] Paez KA (2014). Development of the Health insurance literacy measure (HILM): conceptualizing and measuring consumer ability to choose and use private health insurance. J Health Commun.

[35] Holst L (2022). Health insurance literacy in the Netherlands: the translation and validation of the United States’ Health insurance literacy measure (HILM). PLoS ONE.

[36] Brazier JE (1992). Validating the SF-36 health survey questionnaire: new outcome measure for primary care. BMJ.

[37] Hibbard JH (2004). Development of the patient activation measure (PAM): conceptualizing and measuring activation in patients and consumers. Health Serv Res.

[38] Diviani N (2015). Low health literacy and evaluation of online health information: a systematic review of the literature. J Med Internet Res.

[39] Nakayama K (2022). Associations between health literacy and information-evaluation and decision-making skills in japanese adults. BMC Public Health.

[40] Kim SH, Utz S (2018). Association of health literacy with health information-seeking preference in older people: a correlational, descriptive study. Nurs Health Sci.

[41] Nijman J (2014). Patient activation and health literacy as predictors of health information use in a general sample of dutch health care consumers. J Health Commun.

