# Supplementary material for: The role of health insurance literacy in the process and outcomes of choosing a health insurance policy in the Netherlands
Source: BMC Health Serv Res. 2023 Sep 18;23:1002. doi: 10.1186/s12913-023-09960-0 (PMC10506200; doi:10.1186/s12913-023-09960-0)
Supplement: Supplementary file 1 — Supplementary Material 1 [file 12913_2023_9960_MOESM1_ESM.docx]

**APPENDIX**

| **Table A1: Questions from the monitor of ‘switching health insurer’ in order to answer the main research questions of this study** | | |
| --- | --- | --- |
| **Main research question** | **Questions from the monitor** | **Answer options** |
| Does the HIL level affect the experience of choosing a health insurance policy? | Q1: What do you think about choosing a health insurance policy? Choosing a policy is … | Five ordinal scales were presented with a range from 1 to 5. Scale 1: Difficult (score 1) – Easy (score 5). Scale 2: Not interesting (score 1) – Interesting (score 5). Scale 3: Boring (score 1) – Fascinating (score 5). Scale 4: Important (score 1) – Not important (score 5). Scale 5: Worthwhile (score 1) – Not worthwhile (score 5). |
|  | Q2: During the last switching period (end of 2019), to what extent have you thought about ... 1) … choosing a basic insurance policy? 2) … whether or not to opt for a voluntary deductible? 3) … whether or not to choose a supplementary insurance policy? | Very superficial (score 1); Superficial (score 2); Not superficial/not thoroughly (score 3); Thoroughly (score 4); Very thoroughly (score 5); I have not thought about this (score 6). |
|  | Q3: For the statements below, please indicate to what extent you agree with the statement? 1) I spend a lot of time looking for the right information about health insurance policies. 2) I would like more help with how to find the right information about health insurance policies. | Totally disagree (score 1); Disagree (score 2); Not disagree/not agree (score 3); Agree (score 4); Totally agree (score 5). |
|  | Q4: To what extent are you convinced that you have accessed sufficient information to make a well-informed decision concerning a health insurance policy? | Not convinced at all (score 1); A little convinced (score 2); Reasonably convinced (score 3); Very convinced (score 4). |
| How does the HIL level relate to the behaviour of citizens in switching health insurance policy? | Q5: Have you switched health insurers as of 2020? | I did not switch but I did consider it (score 1); I did not switch and I did not consider it (score 2); I only switched the basic insurance policy (score 3); I only switched the supplementary insurance policy (score 4); I switched both the basic and supplementary insurance policy (score 5). |
|  | Q6: How often did you switch health insurers in the past five years? | Not once (score 1); Once (score 2); Two times (score 3); Three times (score 4); Four times (score 5); Five times (score 6). |
| How does the HIL level affect the choice of health insurance? | Q7: How are you insured in 2020? | Only a basic insurance policy (score 1); A basic and supplementary insurance policy, with additional dental insurance (score 2); A basic and supplementary insurance policy, without additional dental insurance (score 3); A basic insurance policy and additional dental insurance, without another supplementary insurance policy (score 4). |
|  | Q8: Do you have a voluntary deductible on top of your mandatory deductible of €385 this year (2020)? | No (score 1); Yes, namely €100 (score 2); Yes, namely €200 (score 3); Yes, namely €300 (score 4); Yes, namely €400 (score 5); Yes, namely €500 (score 6). |

* The six answer options of question 2 were categorised into ‘very superficial, or superficial ’ (score 1 and 2) ‘not superficial/not thoroughly’ (score 3), ‘thoroughly, or very thoroughly’ (score 4 and 5), and ‘I have not thought about this’ (score 6). The five answer options of question 5 were categorised into ‘not switched’ (1 and 2) and ‘switched’ (3, 4, and 5); the six answer options of question 6 were categorised into ‘not once’ (1) and ‘one time or more’ (2, 3, 4, 5, and 6); the four answer options of question 7 were categorised into ‘a basic insurance policy only’ (1) and ‘both a basic and a supplementary insurance policy’ (2, 3, and 4); and the six answer options of question 8 were categorised into ‘no’ (1) and ‘yes’ (2, 3, 4, 5, and 6).

**Table A2: Ordinal logistic regression analysis to examine the association between the extent to which respondents find choosing a health insurance policy is difficult, interesting, boring, important, or worthwhile, and the demographics included**

|  | | Model 1: Dependent variable: Choosing a policy is difficult (1) - easy (5)   n=699 | | Model 2: Dependent variable: Choosing a policy is not interesting (1) - interesting (5)   n=672 | | | Model 3: Dependent variable: Choosing a  policy is boring (1) - fascinating (5)   n=670 | | Model 4: Dependent variable: Choosing a policy is important (1) – not important (5)   n=689 | | | Model 5: Dependent variable: Choosing a policy is worthwhile (1) – not worthwhile (5)   n=681 | |
| --- | --- | --- | --- | --- | --- | --- | --- | --- | --- | --- | --- | --- | --- |
|  | | Odds ratio | P-value | | Odds ratio | P-value | Odds ratio | P-value | Odds ratio | P-value | | Odds ratio | P-value |
| HIL | Low | ref |  | | ref |  | ref |  | ref | |  | ref |  |
|  | Intermediate | 2.97 | **0.00** | | 0.49 | **0.00** | 1.62 | **0.01** | 0.61 | | **0.02** | 1.79 | **0.00** |
|  | High | 9.15 | **0.00** | | 0.41 | **0.00** | 2.95 | **0.00** | 0.50 | | **0.00** | 2.45 | **0.00** |
| Gender | Male | ref |  | | ref |  | ref |  | ref | |  | ref |  |
|  | Female | 0.72 | **0.03** | | 0.91 | 0.55 | 1.08 | 0.63 | 0.64 | | **0.01** | 1.16 | 0.33 |
| Age | 18-39 | ref |  | | ref |  | ref |  | ref | |  | ref |  |
|  | 40-64 | 1.10 | 0.66 | | 1.12 | 0.60 | 1.11 | 0.62 | 2.24 | | **0.00** | 0.66 | 0.06 |
|  | 65 and older | 1.07 | 0.79 | | 0.90 | 0.68 | 1.17 | 0.54 | 2.37 | | **0.00** | 0.55 | **0.02** |
| Highest completed education level* | Low | ref |  | | ref |  | ref |  | ref | |  | ref |  |
|  | Intermediate | 1.09 | 0.76 | | 0.60 | 0.06 | 1.44 | 0.20 | 0.59 | | 0.06 | 1.35 | 0.28 |
|  | High | 1.03 | 0.91 | | 0.99 | 0.97 | 0.68 | 0.19 | 0.47 | | **0.02** | 1.30 | 0.37 |
| Household net income per month in euros | < 1.750 | ref |  | | ref |  | ref |  | ref | |  | ref |  |
|  | 1.750 - 2.700 | 0.66 | **0.05** | | 1.00 | 1.00 | 1.13 | 0.57 | 0.73 | | 0.18 | 0.95 | 0.81 |
|  | > 2.700 | 0.92 | 0.69 | | 1.42 | 0.10 | 1.00 | 0.98 | 1.14 | | 0.59 | 0.86 | 0.49 |
| Self-reported health | Bad / fair | ref |  | | ref |  | ref |  | ref | |  | ref |  |
|  | Good | 1.13 | 0.63 | | 0.72 | 0.19 | 1.22 | 0.41 | 0.94 | | 0.83 | 1.26 | 0.34 |
|  | Very good / excellent | 1.62 | 0.10 | | 0.81 | 0.46 | 0.89 | 0.70 | 0.97 | | 0.93 | 1.12 | 0.70 |
| Self-reported amount of care used | None | ref |  | | ref |  | ref |  | ref | |  | ref |  |
|  | Very little / little | 0.56 | **0.05** | | 1.36 | 0.28 | 0.70 | 0.22 | 1.55 | | 0.22 | 0.64 | 0.14 |
|  | Much / very much | 0.48 | **0.04** | | 1.29 | 0.47 | 1.12 | 0.74 | 1.44 | | 0.39 | 0.93 | 0.84 |

* Low = none, primary school or pre-vocational education. Intermediate = secondary or vocational education. High = professional higher or university.

**Table A3.1: Multinomial logistic regression analysis to examine the association between the extent to which respondents thought about choosing a basic insurance policy, and the demographics included**

|  | | Model 1: Dependent variable: During the last switching period (end of 2019), to what extent have you thought about ...  1) … choosing a basic insurance policy?  n=704 | | | | | | |
| --- | --- | --- | --- | --- | --- | --- | --- | --- |
|  | | (0 = I have not thought about this, 1 = superficial or very superficial), | | (0 = I have not thought about this, 1 = not superficial not thoroughly), | | | (0 = I have not thought about this, 1 = thoroughly or very thoroughly), | |
|  | | RRR** | P-value | | RRR | P-value | RRR | P-value |
| HIL | Low | ref |  | | ref |  | ref |  |
|  | Intermediate | 1.00 | 0.99 | | 1.50 | 0.14 | 1.99 | **0.02** |
|  | High | 0.55 | **0.03** | | 1.00 | 0.99 | 2.43 | **0.00** |
| Gender | Male | ref |  | | ref |  | ref |  |
|  | Female | 0.66 | 0.05 | | 0.77 | 0.25 | 1.03 | 0.90 |
| Age | 18-39 | ref |  | | ref |  | ref |  |
|  | 40-64 | 0.48 | **0.02** | | 0.41 | **0.01** | 0.38 | **0.00** |
|  | 65 and older | 0.47 | **0.03** | | 0.47 | **0.04** | 0.29 | **0.00** |
| Highest completed education level* | Low | ref |  | | ref |  | ref |  |
|  | Intermediate | 0.75 | 0.42 | | 1.22 | 0.61 | 1.27 | 0.53 |
|  | High | 1.48 | 0.30 | | 0.84 | 0.67 | 1.17 | 0.70 |
| Household net income per month in euros | < 1.750 | ref |  | | ref |  | ref |  |
|  | 1.750 - 2.700 | 1.04 | 0.90 | | 1.12 | 0.71 | 1.29 | 0.39 |
|  | > 2.700 | 0.98 | 0.94 | | 1.26 | 0.47 | 1.23 | 0.49 |
| Self-reported health | Bad / fair | ref |  | | ref |  | ref |  |
|  | Good | 1.08 | 0.82 | | 2.11 | 0.05 | 1.07 | 0.85 |
|  | Very good / excellent | 1.43 | 0.36 | | 1.92 | 0.14 | 0.82 | 0.62 |
| Self-reported amount of care used | None | ref |  | | ref |  | ref |  |
|  | Very little / little | 0.86 | 0.70 | | 1.76 | 0.29 | 0.54 | 0.10 |
|  | Much / very much | 0.84 | 0.72 | | 2.09 | 0.23 | 0.52 | 0.17 |
| Constant |  | 1.51 | 0.54 | | 0.25 | 0.09 | 0.99 | 0.99 |

* Low = none, primary school or pre-vocational education. Intermediate = secondary or vocational education. High = professional higher or university.

** RRR = relative risk ratio

**Table A3.2: Multinomial logistic regression analysis to examine the association between the extent to which respondents thought about whether or not to opt for a voluntary deductible, and the demographics included**

|  | | Model 1: Dependent variable: During the last switching period (end of 2019), to what extent have you thought about ...  2) … whether or not to opt for a voluntary deductible?  n=698 | | | | | | |
| --- | --- | --- | --- | --- | --- | --- | --- | --- |
|  | | (0 = I have not thought about this, 1 = superficial or very superficial), | | (0 = I have not thought about this, 1 = not superficial not thoroughly), | | | (0 = I have not thought about this, 1 = thoroughly or very thoroughly), | |
|  | | RRR** | P-value | | RRR | P-value | RRR | P-value |
| HIL | Low | ref |  | | ref |  | ref |  |
|  | Intermediate | 1.27 | 0.39 | | 1.13 | 0.66 | 1.55 | 0.11 |
|  | High | 0.90 | 0.70 | | 0.70 | 0.24 | 2.04 | **0.01** |
| Gender | Male | ref |  | | ref |  | ref |  |
|  | Female | 0.84 | 0.43 | | 0.67 | 0.09 | 0.61 | **0.02** |
| Age | 18-39 | ref |  | | ref |  | ref |  |
|  | 40-64 | 0.69 | 0.25 | | 0.53 | 0.06 | 0.40 | **0.00** |
|  | 65 and older | 0.57 | 0.14 | | 0.53 | 0.10 | 0.31 | **0.00** |
| Highest completed education level* | Low | ref |  | | ref |  | ref |  |
|  | Intermediate | 1.53 | 0.41 | | 0.61 | 0.18 | 1.12 | 0.77 |
|  | High | 2.98 | **0.04** | | 0.61 | 0.22 | 1.13 | 0.77 |
| Household net income per month in euros | < 1.750 | ref |  | | ref |  | ref |  |
|  | 1.750 - 2.700 | 1.22 | 0.55 | | 0.94 | 0.83 | 1.36 | 0.30 |
|  | > 2.700 | 1.34 | 0.37 | | 1.05 | 0.88 | 1.50 | 0.18 |
| Self-reported health | Bad / fair | ref |  | | ref |  | ref |  |
|  | Good | 1.90 | 0.09 | | 1.89 | 0.12 | 1.33 | 0.40 |
|  | Very good / excellent | 2.24 | 0.07 | | 2.26 | 0.08 | 1.84 | 0.12 |
| Self-reported amount of care used | None | ref |  | | ref |  | ref |  |
|  | Very little / little | 0.59 | 0.22 | | 0.61 | 0.27 | 0.58 | 0.17 |
|  | Much / very much | 0.58 | 0.30 | | 0.44 | 0.14 | 0.49 | 0.14 |
| Constant |  | 0.21 | 0.05 | | 1.08 | 0.91 | 0.80 | 0.74 |

* Low = none, primary school or pre-vocational education. Intermediate = secondary or vocational education. High = professional higher or university.

** RRR = relative risk ratio

**Table A3.3: Multinomial logistic regression analysis to examine the association between the extent to which respondents thought about whether or not to choose a supplementary insurance policy, and the demographics included**

|  | | Model 1: Dependent variable: During the last switching period (end of 2019), to what extent have you thought about ...  3) … whether or not to choose a supplementary insurance policy?  n=707 | | | | | | |
| --- | --- | --- | --- | --- | --- | --- | --- | --- |
|  | | (0 = I have not thought about this, 1 = superficial or very superficial), | | (0 = I have not thought about this, 1 = not superficial not thoroughly), | | | (0 = I have not thought about this, 1 = thoroughly or very thoroughly), | |
|  | | RRR** | P-value | | RRR | P-value | RRR | P-value |
| HIL | Low | ref |  | | ref |  | ref |  |
|  | Intermediate | 1.24 | 0.45 | | 1.54 | 0.15 | 1.56 | 0.06 |
|  | High | 0.78 | 0.41 | | 0.84 | 0.58 | 1.81 | **0.01** |
| Gender | Male | ref |  | | ref |  | ref |  |
|  | Female | 0.79 | 0.31 | | 0.62 | 0.06 | 0.91 | 0.60 |
| Age | 18-39 | ref |  | | ref |  | ref |  |
|  | 40-64 | 0.42 | **0.01** | | 0.62 | 0.23 | 0.41 | **0.00** |
|  | 65 and older | 0.41 | **0.02** | | 0.85 | 0.71 | 0.31 | **0.00** |
| Highest completed education level* | Low | ref |  | | ref |  | ref |  |
|  | Intermediate | 0.80 | 0.60 | | 1.24 | 0.61 | 1.35 | 0.36 |
|  | High | 1.61 | 0.28 | | 0.80 | 0.63 | 1.39 | 0.36 |
| Household net income per month in euros | < 1.750 | ref |  | | ref |  | ref |  |
|  | 1.750 - 2.700 | 1.37 | 0.36 | | 0.88 | 0.71 | 1.45 | 0.14 |
|  | > 2.700 | 1.27 | 0.49 | | 1.24 | 0.54 | 1.23 | 0.44 |
| Self-reported health | Bad / fair | ref |  | | ref |  | ref |  |
|  | Good | 1.14 | 0.74 | | 2.10 | 0.09 | 1.64 | 0.09 |
|  | Very good / excellent | 1.29 | 0.57 | | 2.24 | 0.11 | 1.63 | 0.16 |
| Self-reported amount of care used | None | ref |  | | ref |  | ref |  |
|  | Very little / little | 0.54 | 0.15 | | 1.24 | 0.70 | 0.73 | 0.41 |
|  | Much / very much | 0.27 | **0.02** | | 0.87 | 0.83 | 0.75 | 0.51 |
| Constant |  | 1.35 | 0.69 | | 0.26 | 0.13 | 0.96 | 0.95 |

* Low = none, primary school or pre-vocational education. Intermediate = secondary or vocational education. High = professional higher or university.

** RRR = relative risk ratio

**Table A4: Ordinal logistic regression analysis to examine the association between the extent to which respondents agreed with two statements, and the demographics included**

|  | | Model 1: Dependent variable: I spend a lot of time looking for the right information about health insurance policies.    Totally disagree (score 1); Disagree (score 2); Not disagree/not agree (score 3); Agree (score 4); Totally agree (score 5)   n=692 | | Model 2: Dependent variable: I would like more help with how to find the right information about health insurance policies  Totally disagree (score 1); Disagree (score 2); Not disagree/not agree (score 3); Agree (score 4); Totally agree (score 5)   n=693 | | |
| --- | --- | --- | --- | --- | --- | --- |
|  | | Odds ratio | P-value | | Odds ratio | P-value |
| HIL | Low | ref |  | | ref |  |
|  | Intermediate | 2.40 | **0.00** | | 0.47 | **0.00** |
|  | High | 2.49 | **0.00** | | 0.23 | **0.00** |
| Gender | Male | ref |  | | ref |  |
|  | Female | 1.14 | 0.34 | | 1.14 | 0.37 |
| Age | 18-39 | ref |  | | ref |  |
|  | 40-64 | 0.61 | **0.01** | | 1.01 | 0.97 |
|  | 65 and older | 0.44 | **0.00** | | 1.59 | **0.04** |
| Highest completed education level* | Low | ref |  | | ref |  |
|  | Intermediate | 1.10 | 0.70 | | 0.73 | 0.22 |
|  | High | 0.77 | 0.33 | | 0.44 | **0.00** |
| Household net income per month in euros | < 1.750 | ref |  | | ref |  |
|  | 1.750 - 2.700 | 0.97 | 0.89 | | 1.34 | 0.13 |
|  | > 2.700 | 0.81 | 0.30 | | 0.72 | 0.11 |
| Self-reported health | Bad / fair | ref |  | | ref |  |
|  | Good | 1.33 | 0.21 | | 0.98 | 0.93 |
|  | Very good / excellent | 0.99 | 0.98 | | 0.57 | **0.04** |
| Self-reported amount of care used | None | ref |  | | ref |  |
|  | Very little / little | 0.50 | **0.01** | | 0.96 | 0.87 |
|  | Much / very much | 0.70 | 0.27 | | 0.89 | 0.73 |

* Low = none, primary school or pre-vocational education. Intermediate = secondary or vocational education. High = professional higher or university.

**Table A5: Ordinal logistic regression analysis to examine the association between the extent to which respondents indicated they were convinced that they have accessed sufficient information to make a well-informed decision concerning a health insurance policy, and the demographics included**

|  | | Model 1: Dependent variable: Convinced    Not convinced at all (score 1); A little convinced (score 2); Reasonably convinced (score 3); Very convinced (score 4).  n=710 | |
| --- | --- | --- | --- |
|  | | Odds ratio | P-value |
| HIL | Low | ref |  |
|  | Intermediate | 4.07 | **0.00** |
|  | High | 21.37 | **0.00** |
| Gender | Male | ref |  |
|  | Female | 1.02 | 0.89 |
| Age | 18-39 | ref |  |
|  | 40-64 | 0.65 | 0.05 |
|  | 65 and older | 0.49 | **0.00** |
| Highest completed education level* | Low | ref |  |
|  | Intermediate | 0.87 | 0.60 |
|  | High | 1.04 | 0.89 |
| Household net income per month in euros | < 1.750 | ref |  |
|  | 1.750 - 2.700 | 1.08 | 0.71 |
|  | > 2.700 | 1.04 | 0.85 |
| Self-reported health | Bad / fair | ref |  |
|  | Good | 1.03 | 0.90 |
|  | Very good / excellent | 1.18 | 0.57 |
| Self-reported amount of care used | None | ref |  |
|  | Very little / little | 0.83 | 0.53 |
|  | Much / very much | 0.84 | 0.62 |

* Low = none, primary school or pre-vocational education. Intermediate = secondary or vocational education. High = professional higher or university.

**Table A6: Dichotomous logistic regression analysis to examine the association between switching and the demographics included**

|  | | Model 1: Dependent variable: Have you switched health insurers as of 2020? (0=no, 1=yes)   n=715 | | | Model 2: Dependent variable: How often did you switch health insurers in the past five years? (0=none, 1=one time or more)   n=712 | | |
| --- | --- | --- | --- | --- | --- | --- | --- |
|  | | | Odds ratio | P-value | | Odds ratio | P-value |
| HIL | Low | | reference |  | | reference |  |
|  | Intermediate | | 0.96 | 0.94 | | 1.13 | 0.60 |
|  | High | | 3.27 | **0.00** | | 1.74 | **0.02** |
| Gender | Male | | reference |  | | reference |  |
|  | Female | | 0.94 | 0.84 | | 1.04 | 0.84 |
| Age | 18-39 | | reference |  | | reference |  |
|  | 40-64 | | 0.41 | **0.02** | | 0.24 | **0.00** |
|  | 65 and older | | 0.29 | **0.01** | | 0.15 | **0.00** |
| Highest completed education level* | Low | | reference |  | | reference |  |
|  | Intermediate | | 1.83 | 0.35 | | 0.77 | 0.42 |
|  | High | | 2.04 | 0.30 | | 1.16 | 0.67 |
| Household net income per month in euros | < 1.750 | | reference |  | | reference |  |
|  | 1.750 - 2.700 | | 0.72 | 0.42 | | 0.74 | 0.25 |
|  | > 2.700 | | 0.41 | **0.04** | | 0.71 | 0.19 |
| Self-reported health | Bad / fair | | reference |  | | reference |  |
|  | Good | | 1.22 | 0.68 | | 0.76 | 0.36 |
|  | Very good / excellent | | 0.84 | 0.77 | | 0.74 | 0.38 |
| Self-reported amount of care used | None | | reference |  | | reference |  |
|  | Very little / little | | 0.68 | 0.52 | | 0.53 | 0.05 |
|  | Much / very much | | 1.15 | 0.84 | | 0.36 | **0.02** |
| Constant |  | | 0.09 | 0.02 | | 3.10 | 0.05 |

* Low = none, primary school or pre-vocational education. Intermediate = secondary or vocational education. High = professional higher or university.

**Table A7: Dichotomous logistic regression analysis to examine the association between health insurance choices and the demographics included**

|  | | Model 1: Dependent variable: How are you insured in 2020? (0= basic insurance policy only, 1= both a basic and a supplementary insurance policy)   n=714 | | | Model 2: Dependent variable: Do you have a voluntary deductible on top of your mandatory deductible of €385 this year (2020)?  (0=no, 1=yes)   n=714 | | |
| --- | --- | --- | --- | --- | --- | --- | --- |
|  | | | Odds ratio | P-value | | Odds ratio | P-value |
| HIL | Low | | reference |  | | reference |  |
|  | Intermediate | | 0.79 | 0.43 | | 0.76 | 0.35 |
|  | High | | 0.59 | 0.07 | | 0.80 | 0.43 |
| Gender | Male | | reference |  | | reference |  |
|  | Female | | 1.73 | **0.02** | | 0.42 | **0.00** |
| Age | 18-39 | | reference |  | | reference |  |
|  | 40-64 | | 2.55 | **0.00** | | 0.36 | **0.00** |
|  | 65 and older | | 1.38 | 0.32 | | 0.18 | **0.00** |
| Highest completed education level* | Low | | reference |  | | reference |  |
|  | Intermediate | | 0.32 | **0.04** | | 2.27 | 0.20 |
|  | High | | 0.18 | **0.00** | | 4.93 | **0.01** |
| Household net income per month in euros | < 1.750 | | reference |  | | reference |  |
|  | 1.750 - 2.700 | | 1.67 | 0.10 | | 1.08 | 0.83 |
|  | > 2.700 | | 2.23 | **0.01** | | 0.86 | 0.66 |
| Self-reported health | Bad / fair | | reference |  | | reference |  |
|  | Good | | 1.35 | 0.43 | | 3.79 | 0.05 |
|  | Very good / excellent | | 1.61 | 0.27 | | 5.61 | **0.01** |
| Self-reported amount of care used | None | | reference |  | | reference |  |
|  | Very little / little | | 2.98 | **0.00** | | 0.61 | 0.16 |
|  | Much / very much | | 6.01 | **0.00** | | 0.15 | **0.00** |
| Constant |  | | 2.03 | 0.36 | | 0.13 | 0.04 |

* Low = none, primary school or pre-vocational education. Intermediate = secondary or vocational education. High = professional higher or university.
